# Adenovirus 5 Vectors Expressing SARS-CoV-2 Proteins

**DOI:** 10.1128/msphere.00998-21

**Published:** 2022-02-28

**Authors:** Scott Bachus, Nikolas Akkerman, Lauren Fulham, Drayson Graves, Caelan Stephanson, Harram Memon, Matthew S. Miller, Peter Pelka

**Affiliations:** a Department of Microbiology, University of Manitoba, Winnipeg, Manitoba, Canada; b Michael G. DeGroote Institute for Infectious Disease Research, McMaster University, Hamilton, Ontario, Canada; c Department of Medical Microbiology and Infectious Diseases, University of Manitoba, Winnipeg, Manitoba, Canada; University of Michigan-Ann Arbor

**Keywords:** COVID-19, SARS-CoV-2, adenovirus, viral vectors

## Abstract

SARS-CoV-2 coronavirus is a recently identified novel coronavirus that is the causative agent of the COVID-19 pandemic that began in 2020. An intense research effort has been undertaken by the research community in order to better understand the molecular etiology of this virus and its mechanisms of host cell subjugation and immune system evasion. To facilitate further research into the SARS-CoV-2 coronavirus we have generated adenovirus 5-based viral vectors that express SARS-CoV-2 proteins—S, N, E, NSP7, NSP8, and NSP12 as hemagglutinin (HA)-tagged and untagged variants. We have also engineered two additional viruses that express the S protein receptor binding domain and a fusion of the receptor binding domain to the N protein. We show that these vectors are expressed in several different cell lines by Western blotting and real-time quantitative reverse transcriptase (qRT-PCR), we evaluate the subcellular localization of these viral proteins, and we show that these coronavirus proteins bind to a variety of cellular targets. The flexibility of adenovirus vectors allows them to be used in a variety of cell models and, importantly, in animal models as well.

**IMPORTANCE** The COVID-19 pandemic caused by the SARS-CoV-2 coronavirus has brought untold personal and economic suffering to the world. Intense research has made tremendous progress in understanding how this virus works, yet much research remains to be done as new variants and continued evolution of the virus keep shifting the rules of engagement on the pandemic battlefield. Therefore, wide availability of resources and reagents to study SARS-CoV-2 is essential in overcoming the pandemic and for the prevention of future outbreaks. Our viral vectors provide additional tools for researchers to use in order to better understand the molecular biology of virus-host interactions and other aspects of SARS-CoV-2.

## INTRODUCTION

SARS-CoV-2 is the causative agent of COVID-19 (1, 2). The virus belongs to the *Coronaviridae* family within the order *Nidovirales*, initially identified in the 1930s as the causative agent of bronchitis in chickens, pig gastroenteritis, and hepatitis and neurological disease in mice (3). These viruses are a large family of pathogens that infect mammals and birds, responsible for a variety of diseases, including COVID-19, Middle East respiratory syndrome (MERS), severe acute respiratory syndrome (SARS), and less-severe ailments such as minor colds and gastroenteritis (3, 4). The virions of coronaviruses are spherical and enveloped, ranging in diameter from 120 to 160 nm, with the spike protein forming a large (∼20-nm) structure on electron microscopy images, reminiscent of the solar corona, giving these viruses their name (3). The coronavirus nucleocapsid is helical and contains the large viral RNA genome (3). The viral genome is a single-stranded positive-sense RNA with a 5′ cap and 3′ polyA tail; this genome alone is infectious and can be directly translated by the host ribosome to make the precursor polyprotein that forms the replicase and smaller proteins translated separately (3). The genome is one of the largest for nonsegmented RNA viruses. The genome itself encodes 14 open reading frames that generate 27 different proteins (5), including the surface glycoprotein S, the membrane glycoprotein M, envelope protein E, the RNA-dependent RNA polymerase (RdRP), the nucleocapsid protein N, and several other accessory proteins. In total, the virus produces 15 nonstructural proteins, 8 accessory proteins, and 4 structural proteins (5, 6). The pandemic caused by SARS-CoV-2 has killed countless individuals, caused indescribable suffering, and left massive economic devastation across the world (7). Therefore, there continues to be a need for tools to further study these viruses.

Human adenovirus (HAdV) was initially identified as the causative agent of cell degeneration during attempts to culture epithelial-like cells from adenoid tissue surgically removed from children (8). HAdVs offer several unique and useful features that make them attractive for the development of vaccines. HAdVs are widely distributed among the human population and generally cause mild disease. Of these, species C viruses, widely used for vaccine development (9), are the best studied and understood (3). This makes them particularly attractive for viral vector design due to widely available tools for recombineering, guaranteed infectivity in humans and a wide range of animals, extensive cellular tropism, and well-established manufacturing methods. The HAdV genome is comparatively large, allowing for insertion of large genes or multiple sequences of >30,000 base pairs in completely gutted vectors (10). Importantly, there exists a large variety of reagents and vectors for viral recombineering, making engineering of novel vectors a relatively straightforward and rapid process (10). This easy recombineering allows for generation of both replication-competent and replication-incompetent vectors, depending on the desired need (9). Furthermore, not only is HAdV a stable virus that is easy to manipulate in the lab, but HAdV can be easily grown and scaled up in industrial settings for rapid vaccine scaleup and deployment when needed. Importantly, several vaccines for COVID-19 have been developed with the use of adenovirus as a vector, including vaccines from Johnson & Johnson, AstraZeneca, and Sinovac, the Sputnik V vaccine, and others; for a comprehensive list see reference 7. Despite exhibiting strict host-range restrictions for productive infection, HAdV is able to infect and deliver genes to a wide variety of hosts and cell types, making *in vivo* studies using different animals feasible.

In the present study, we report on the design, construction, and validation of multiple HAdV5-based viral vectors that express several different SARS-CoV-2 proteins. We decided to make vectors expressing proteins that would be of the most interest from a clinical perspective, including those that are good antigen candidates (structural components) or those that can be targeted for drug development (RdRP). We show that these proteins can be immunoprecipitated from infected cells, and we investigate their subcellular localization. These vectors were designed to be freely available to anyone interested in using them for research purposes in animal and cell models.

## RESULTS

### Design and construction of recombinant HAdVs expressing SARS-CoV-2-genes.

**FIG 1:**
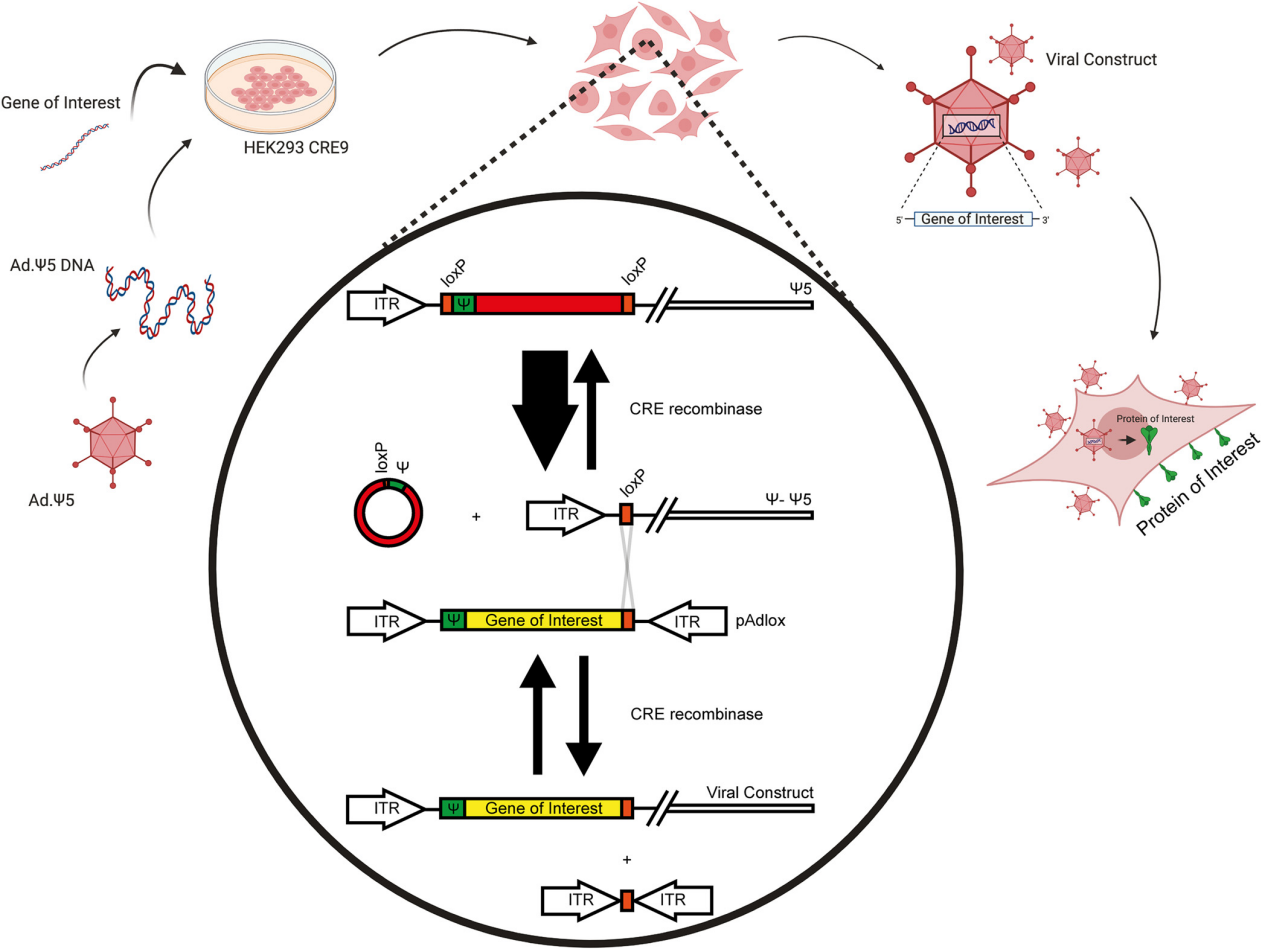
Schematic of Cre-LoxP-based viral recombineering to generate rHAdV vectors. Diagram representing the recombineering strategy for making rHAdV vectors. Modified from reference 11 with permission.

To design recombinant HAdVs (rHAdVs) we used the method of Hardy et al. (11) that utilizes the Cre recombinase together with engineered loxP sites to *in vivo* recombine a transgene of interest into the viral backbone (Fig. 1). A previously described (12, 13) isolate of SARS-CoV-2 was used as the donor for the viral structural and nonstructural genes. SARS-CoV-2 genes (NSP7, NSP8, NSP12, E, N, S receptor binding domain [RBD, or RBD-N fusion) were subcloned into the donor plasmid, pAdLox-P, sequenced, and cotransfected with viral backbone, Ψ5, into 293Cre9 cells using linear polyethylenimine lipid. 293Cre9 cells are a subclone of 293Cre8 cells (14) that have been specifically selected for efficient viral recombination. Two versions of each virus were generated, one carrying a hemagglutinin (HA) tag at the N terminus of the protein and one without the tag, with the exception of the spike gene, which did not recombine correctly when tagged with HA. Viral comets were used to identify recombination, and following two rounds of plaque purification, the viruses were assessed for expression of the gene of interest by Western blotting (for HA-tagged proteins) or quantitative real-time reverse transcriptase PCR (qRT-PCR; for nontagged proteins).

### Expression of SARS-CoV-2 genes in rHAdV vectors.

**FIG 2:**
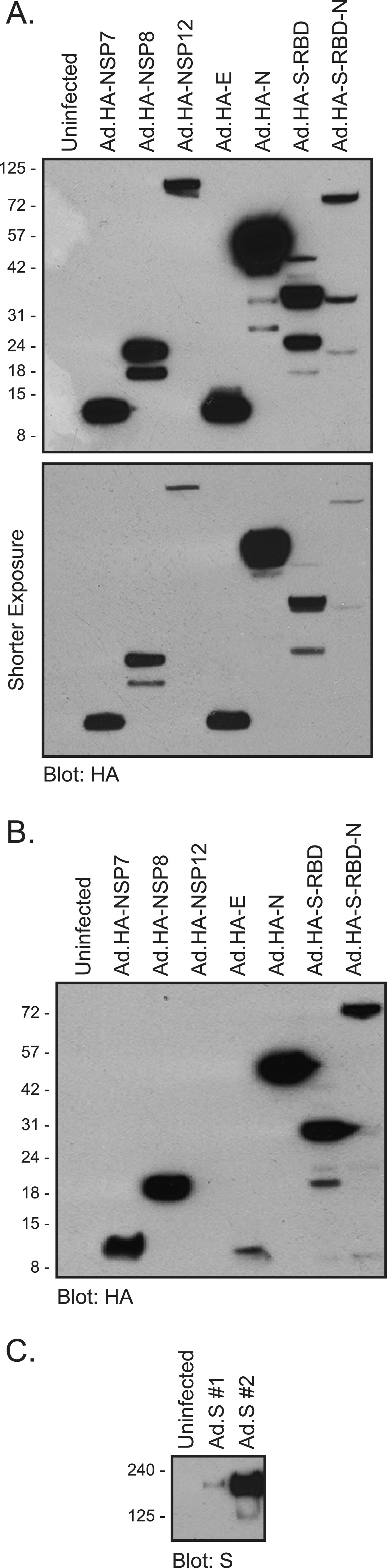
Viral protein expression in 293 and A549 cells infected with the indicated viral constructs. (A) 293 cells were infected with the indicated viruses at an MOI of 500 vp/cell; 24 h after infection cells were harvested and lysed. Then, 30 μg of the total cell lysate was resolved by SDS-PAGE and probed for HA using the rat monoclonal anti-HA antibody. Two exposures are shown, along with the molecular weight marker indicators. The predicted molecular weights in kDa for these proteins are as follows: NSP7, 9.4; NSP8, 22; NSP12, 107; E, 8.4; N, 46; RBD, 31; RBD-N, 76. (B) The same as panel A except A549 cells were infected at an MOI of 1,000 vp/cell for 72 h. (C) The same as panel B except cells were infected with untagged Ad.S at an MOI of 1,000 vp/cell for 72 h and blotted with anti-S antibody 2B3E5. #1 is a construct with a WPRE, whereas #2 does not have this element. The predicted molecular weight of S is 141 kDa.

Expression testing of recombinant viruses was performed in either 293 or A549 cells (Fig. 2). The 293 cells were infected with the rHAdVs expressing the HA-tagged SARS-CoV-2 proteins at a multiplicity of infection (MOI) of 500 viral particles (vp) per cell, while the A549 cells were infected at a 1,000 vp/cell for 72 h. All SARS-CoV-2 proteins that were tagged with HA were readily detectable in the infected 293 cells (Fig. 2A), including the relatively small products arising from NSP7 and E. In A549 cells expression was somewhat lower than in 293 cells (Fig. 2B), and interestingly, we were unable to detect NSP12 despite its robust expression in 293 cells (Fig. 2A). The higher expression in 293 cells was likely the result of amplification due to virus replication in these cells; no replication occurred in A549 cells. There were multiple bands observed for several of the viral proteins, particularly N and NSP8, as well as the S-RBD and S-RBD-N fusion. These appear to correlate with the level of expression, as they were less apparent in A549 cells. We also tested the expression of the nontagged S construct, and it was readily detectable in A549 cells at an MOI of 1,000 vp/cell 72 h after infection (Fig. 2C). We have designed two variants of the vectors expressing untagged S, one (#1) with a woodchuck hepatitis virus posttranscriptional regulatory element (WPRE) and one without (#2). This element is thought to enhance viral transgene expression (15); however, we found that in the context of Ad.S vector, it had the opposite effect and reduced expression of the SARS-CoV-2 S protein.

**FIG 3:**
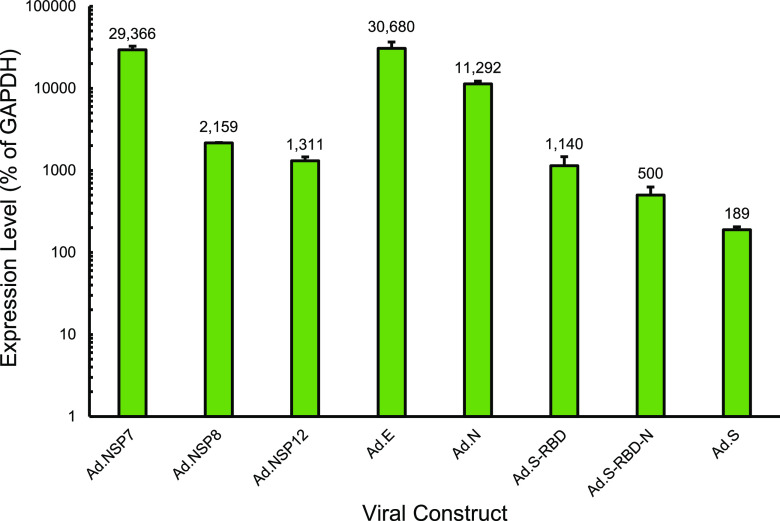
Expression of nontagged SARS-CoV-2 mRNAs encoding SARS-CoV-2 proteins. 293 cells were infected with the indicated rHAdVs at an MOI of 10 vp/cell for 24 h. Total RNA was subsequently extracted using the TRIzol method, and cDNA was transcribed using VILO master mix. Real-time qRT-PCR was performed using primers specific for each gene and SYBR master mix for CFX on a Bio-Rad CFX96 real-time PCR instrument. GAPDH was used as an internal control. Error bars represent the standard deviation of 3 biological replicates. Numbers represent the percentage of GAPDH level.

To test the expression of nontagged constructs, qRT-PCR was used after RNA extraction from infected 293 cells (Fig. 3). Quantification was done relative to GAPDH (glyceraldehyde-3-phosphate dehydrogenase) levels and is represented as the percentage of GAPDH. All nontagged viral construct mRNA was detectable and at very high levels in infected cells (Fig. 3).

### SARS-CoV-2 proteins expressed from rHAdV vector bind to cellular targets.

**FIG 4:**
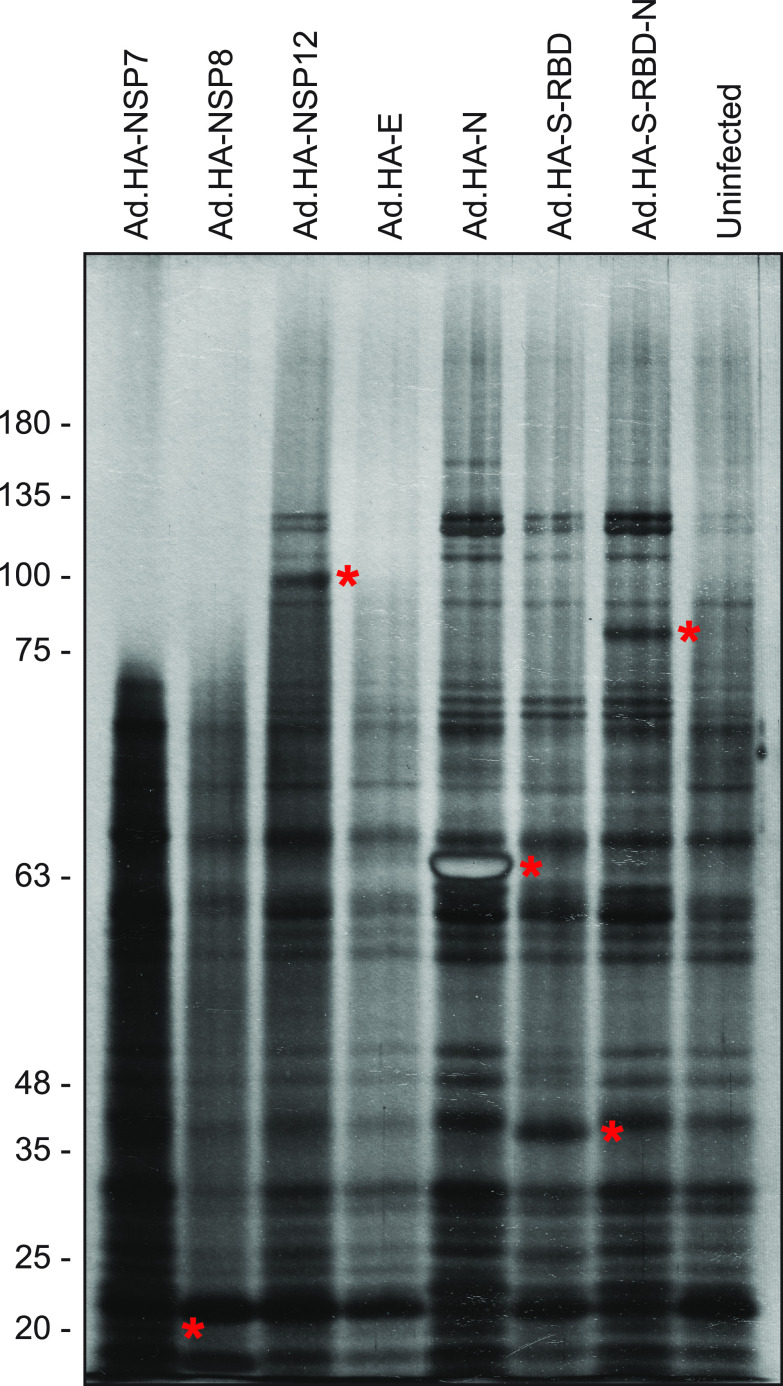
SARS-CoV-2 proteins bind cellular proteins. rHAdV vectors expressing the indicated proteins were infected into 293 cells at an MOI of 100 vp/cell for 24 h. Cells were then harvested, lysed, and immunoprecipitated using anti-HA resin. Proteins were eluted and separated by SDS-PAGE on a 10% polyacrylamide gel, followed by silver staining. HA-tagged SARS-CoV-2 proteins are indicated with asterisks.

We wanted to determine whether the SARS-CoV-2 proteins expressed from rHAdV were able to interact with potential cellular proteins. SARS-CoV-2 proteins were immunoprecipitated from 293 cells infected with rHAdV using anti-HA affinity resin and resolved on SDS-PAGE (Fig. 4). We used uninfected 293 cells as a negative control. Bands from some of the SARS-CoV-2 proteins were detectable in the silver-stained gel (indicated with asterisks in Fig. 4), but some smaller proteins likely did not resolve and were not seen. Nevertheless, we observed a number of unique bands not present in the uninfected sample that suggest the presence of potential binding partners for SARS-CoV-2 proteins. This offers a proof-of-principle that our rHAdV vectors expressing SARS-CoV-2 proteins can be used to study the molecular biology of this virus.

### Subcellular localization of SARS-CoV-2 proteins.

**FIG 5:**
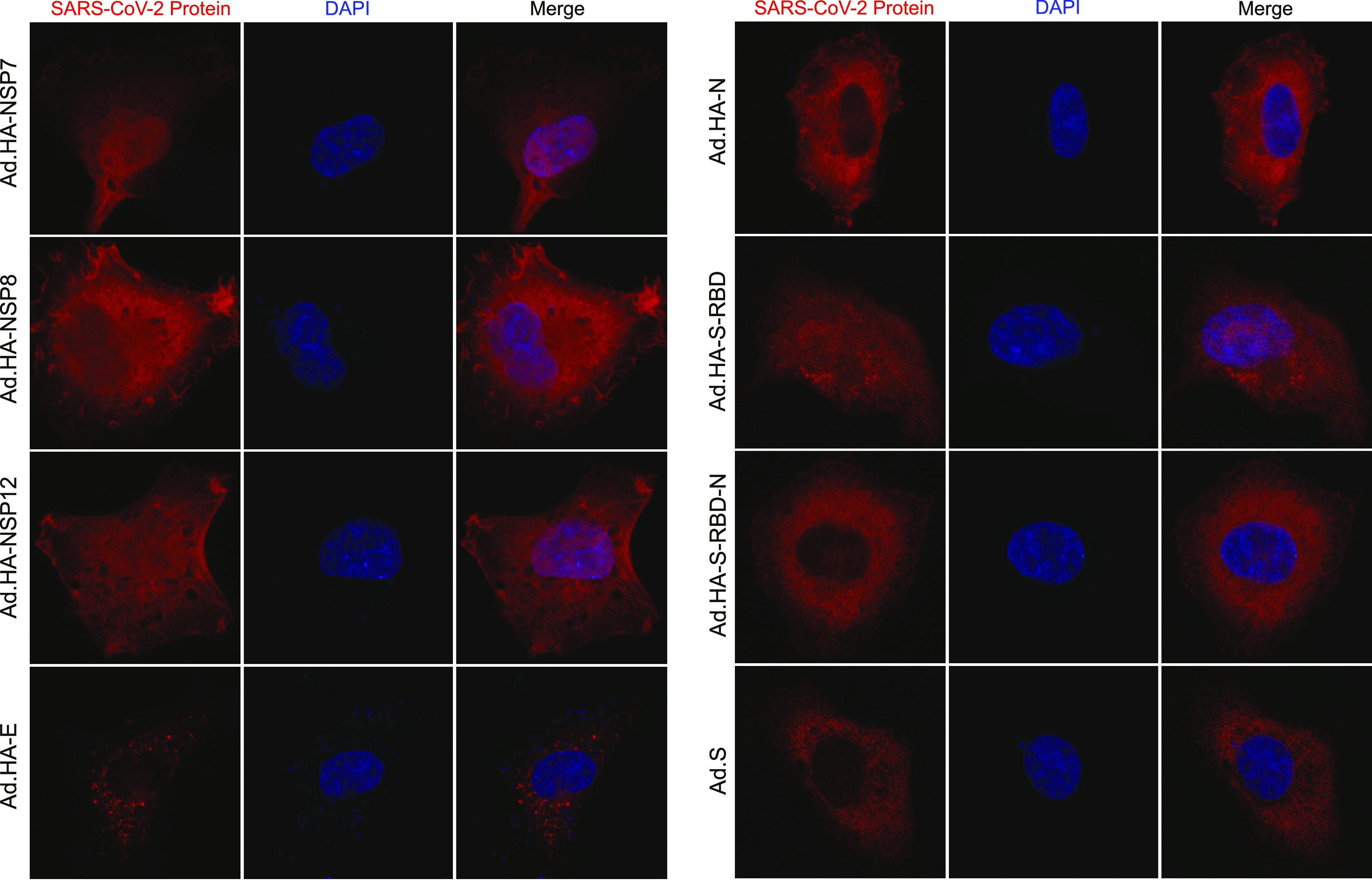
Subcellular localization of SARS-CoV-2 protein expressed from rHAdV vectors. HT1080 cells were infected with the indicated viruses at an MOI of 1,000 vp/cell. Then, 24 h after infection, cells were fixed and stained either for HA using the rat monoclonal 3F10 or for S using 2B3E5. DAPI was used as a nuclear counterstain, and representative images for each protein are shown. Images were acquired on a Zeiss LSM700 laser confocal microscope using a 63× lens objective.

We wanted to determine the subcellular localization of the various SARS-CoV-2 proteins expressed from our rHAdV vectors. To do this, HT1080 cells were infected and stained for the various viral proteins using the anti-HA rat antibody 3F10 (Fig. 5), with the exception of S, which was visualized using an anti-S antibody. All SARS-CoV-2 proteins were detectable in infected HT1080 cells by immunofluorescence. Most of the proteins showed cytoplasmic localization as would be expected of a virus that replicates in the cytoplasm. Interestingly, there were some deviations from this norm. NSP7 showed more pronounced nuclear staining, although it was still present in the cytoplasm. NSP8 and NSP12 showed nucleocytoplasmic staining, while E showed punctate cytoplasmic staining. S-RBD also showed nucleocytoplasmic staining, but the S-RBD-N fusion was predominantly cytoplasmic. Interestingly, S showed predominantly cytoplasmic staining with no plasma membrane accumulation. The presence of the HA epitope tag at the N terminus may interfere with the signal peptide and proper targeting of some proteins, but interestingly, the nontagged S showed a predominantly cytoplasmic localization despite the absence of the HA-tag and the expected targeting of this protein to the plasma membrane. Together, these results show that our rHAdVs express SARS-CoV-2 proteins in HT1080 cells and that these can be visualized using the HA-tag (except for S, which was visualized using anti-S antibody since it was untagged).

## DISCUSSION

In the present study, we report the design, construction, and characterization of rHAdV vectors expressing several different SARS-CoV-2 proteins. We show that all of our viruses express SARS-CoV-2 proteins tagged with an HA-tag, and untagged variants express SARS-CoV-2 mRNA. We further demonstrate the subcellular localization of these proteins, and we show that they interact with a variety of unknown cellular proteins. Together, these viruses represent valuable tools for researchers aiming at understanding SARS-CoV-2 and offer the unique ability of using these not only in an *in vitro* cell culture-based model, but also as tools for animal studies due to the broad range of species that adenoviruses are able to transduce.

The impact of the COVID-19 pandemic on the world has been tremendous, affecting all aspects of society and our way of life (12). This continues to be the case as new variants of SARS-CoV-2 emerge while the world struggles to vaccinate the population (16). Therefore, there continues to be a need for further research in order to better understand and combat the causative agent of COVID-19. The viruses we have generated represent flexible tools for researchers to use in their continued work of understanding SARS-CoV-2, and the flexibility provided by the rHAdV offers a wide range of applications.

We have designed our rHAdV to express either HA-tagged or untagged versions of SARS-CoV-2 proteins, as antibodies, particularly to the NSPs, are not readily available or are of various quality. Therefore, having consistent antigen detection may be important in certain circumstances. Ultimately, having the choice of using either HA-tagged or untagged versions of our viruses provides added flexibility in an assay-dependent manner. Interestingly, recombination of HA-tagged S construct was unsuccessful despite repeated attempts, while untagged S recombined easily. It is unclear why that was the case. It is possible that the combination of the sequences of HA-tag and S gene somehow interfered with the Cre recombinase, or it may be that the HA-tagged S protein itself was somehow toxic and killed recombined cells before efficient virus production was achieved. Ultimately, we felt it was more important to have S in any form, as there is a wide variety of anti-S antibodies, and not having an HA-tagged variant is of limited consequence overall.

Expression of all NSPs and other structural proteins was easily detectable by a Western blot for the HA-tag (Fig. 2) using the high-affinity rat monoclonal antibody 3F10. Several of the proteins showed multiple bands on the Western blot indicative of smaller species; these are likely caused by proteolytic cleavage of the full-length protein, as they are generally more pronounced in proteins that expressed at high levels, such as N. Interestingly, it was puzzling to not see NSP12 express at detectable levels in A549 cells, which are derived from the lung. Some of this is undoubtedly due to a lack of virus replication and overall reduced expression versus 293 cells, but it is possible that stability or other aspects of NSP12 in A549 may play a factor. For example, the fusion protein of S-RBD-N, which expressed similarly to NSP12 in 293 cells, was easily detectable in A549 cells, but NSP12 was not. This may hint of potential regulation of the NSP12 half-life present in one of the cell lines but absent in another and may hint at the potential cellular tropism of the virus itself.

As a proof-of-principle, we performed immunoprecipitation of the HA-tagged SARS-CoV-2 proteins to determine whether they interacted with cellular targets (Fig. 4). Most of the SARS-CoV-2 proteins were readily detectable in the immunoprecipitation resolved on SDS-PAGE and silver-stained, with the exception of the smallest proteins, two of which likely did not resolve (NSP7 and E), and the third had the same molecular weight as a background band observed in all samples, likely masking it (NSP8). These three proteins also had the fewest binding partners; this may be unsurprising for the E protein but was somewhat unexpected for the replicase accessory factors NSP7 and NSP8 (17). Nevertheless, other viral proteins, including the structural N, showed multiple cellular proteins that are bound during infection, providing a potential rich avenue for functional exploration. To further characterize these proteins, we investigated their cellular localization using fluorescence confocal microscopy (Fig. 5). As would be expected of an RNA virus replicating predominantly in the cytoplasm, all of the proteins showed cytoplasmic localization with some variation. In particular, the proteins involved in viral genome replication (NSP7, NSP8, and NSP12) showed a distinct nucleocytoplasmic localization. This may be suggestive of potential nuclear functions of these proteins and perhaps involvement in suppression of antiviral responses or other functions. Indeed, NSP12 has previously been shown to attenuate type I interferon signaling (18). The viral E protein showed a distinct punctate localization, likely due to its association with the endoplasmic reticulum (ER) and the Golgi network (19). The receptor binding domain of S showed a nucleocytoplasmic distribution, whereas the fusion protein S-RBD-N showed cytoplasmic localization only. This is likely due to the small size of S-RBD alone, allowing it to freely cross into the nucleus, something that may also be the case for NSP7 and NSP8. We should note here that for those proteins with an N-terminal signal peptide, the presence of the HA-tag might affect their cellular localization to some degree. Ultimately, these proof-of-principle studies not only provide insights into SARS-CoV-2 protein functions but pave the way for further research.

In conclusion, we have engineered a number of viral vectors based on HAdV5 that individually express a number of SARS-CoV-2 proteins. Characterization of these vectors shows that they express efficiently and provide new tools in the race to understand the virus. Importantly, the flexibility of adenovirus-based vectors enables these to be used in a variety of cell types and animals. These vectors are freely available to anyone interested in their use for their research.

## MATERIALS AND METHODS

### Antibodies.

Rat monoclonal anti-HA 3F10 (MilliporeSigma) was used at a dilution of 1:3,500 for Western blotting. Mouse monoclonal anti-S (MilliporeSigma) was used at a dilution of 1:2,000 for Western blotting. For immunofluorescence, 3F10 rat monoclonal anti-HA was used at a dilution of 1:100, and 2B3E5 was used at a dilution of 1:67. Secondary goat anti-rat and goat anti-mouse horseradish peroxidase (HRP)-conjugated antibodies were purchased from Jackson Immunoresearch and were used at a dilution of 1:100,000 for Western blotting, and Alexa Fluor 594 secondary antibody was purchased from Thermo Fisher Scientific and used at a dilution of 1:600 for immunofluorescence.

### Cells and virus culture.

293Cre9 cells, 293 cells (ATCC CRL-1573), human epithelial lung carcinoma A549 cells (ATCC CRM-CCL-185), and human connective tissue fibrosarcoma HT1080 cells (ATCC CCL-121) were grown in Dulbecco’s modified Eagle’s medium (DMEM) (HyClone) and supplemented with either 10% (293Cre9 and 293) or 5% (A549 and HT-1080) fetal bovine serum (VWR Seradigm) and streptomycin and penicillin (HyClone). Cells were incubated at 37°C under 5% CO_2_. Cotransfections for viral vector recombination were carried out in 10-cm plates of 293Cre9 cells. Complete medium was changed on 293Cre9 plates, and then a transfection mixture containing 1 mL of serum free DMEM, 7 μg of extracted Ψ5 viral DNA, 3 μg of SfiI (New England Biolabs) digested pAdLoxP expression plasmid, and 20 μl of polyethylenimine (PEI) were added. Viruses were allowed to recombine until all cells were dead. All virus infections were carried out in serum-free medium for 1 h, after which, complete medium was added without removal of the infection medium. For all infections, virus titers were determined using viral genome quantification via qPCR, and crude freeze-thawed lysates were used. All recombinant viral vectors were purified by picking plaques from plaque assays performed on 293 cells.

### Cloning.

All genes were amplified using the specific cloning primers described below, using a Phusion PCR kit (New England Biolabs), according to the manufacturer’s specifications. Genes were then cloned into either shuttle vector pAdLoxP and pAdLoxP-WPRE or the expression vector pCAN-HA using standard techniques. pCAN-HA constructs were then subcloned into the pAdLoxP- and pAdLoxP-modified shuttle vectors. Each shuttle vector was then digested using the restriction enzyme SfiI and ethanol extracted, in preparation for cotransfection with Ψ5 viral DNA as previously described.

### PCR primers.

All primers were purchased from Integrated DNA Technologies, and an annealing temperature of 60°C was used.

The cloning PCR primers were the following: NSP7 primers, ACTGTACTCGAGACCATGAGCAAGATGAGCGACGTAAAAT and ACTGTATCTAGATTACTGCAATGTCGCCCGGTTGT; NSP8 primers, ACTGTAGGATCCACCATGGCAATCGCATCTGAATTTTCT and ACTGTAGGATCCTTACTGCAGTTTGACTGCGCTGTTAG; NSP12 primers, ACTGTAGGATCCACCATGTCAGCAGACGCACAAAGTTTTC and ACTGTAGGATCCTTACTGCAGGACGGTGTGAGGCG; E primers, ACTGTAGGATCCACCATGTACAGCTTCGTATCAGAAGAAACC and ACTGTAGGATCCTTAAACGAGGAGATCCGGCACCC; N primers, ACTGTAGGATCCACCATGAGCGATAACGGCCCCCA and ACTGTAGGATCCTTACGCCTGAGTAGAATCGGCTGAG; S-RBD primers, untagged, ACTGCCAAGCTTACCATGGTACAACCTACGGAATCTATCGTACGC and with HA-tag, ACTGCCAAGCTTACCATGGGCTACCCCTACGACGTGCCCGACTACGCCGTACAACCTACGGAATCTATCGTACGC and ACTGCCGGATCCTCCAAATGAGCATGGAGTTATATCG; S primers, ACTGTAGGATCCACCATGTTTGTTTTCTTGGTTCTTTTGCCAC and ACTGTAGGATCCTTACGTGTAGTGCAATTTTACGCCTTTA. The real-time gene expression PCR primers were the following: NSP7 primers, TGAGCAAGATGAGCGACGTA and CATTGACAGGAGAACGCTCA; NSP8 primers, CCGCTCAGAGGATAAACGAG and GAACGTCGTACCATCACACG; NSP12 primers, TGTCAATTTGCATTCCTCCA and TACCGGGTTTTACGGTTTGA; E primers, CCGGGACACTGATCGTAAAT and GGCACCCTGCTAGAATTGAG; N primers, GCATCAGAGGTGGAGATGGT and ACGATTGCGGCATTATTAGC; S-RBD primers, ACTGCCAAGCTTACCGTACAACCTACGGAATCTATCGTACGC and ACTGCCGGATCCTCCAAATGAGCATGGAGTTATATCG; S primer, AACTGAATCGGGCACTTACG CCCAAGACAATCGCCATACT; GAPDH primers, GAGTCAACGGATTTGGTCGT and TTGATTTTGGAGGGATCTCG.

### Western blots.

The 293 and A549 cells were plated, allowed to grow until confluent, and then infected with each viral vector at the indicated specific MOI. Cells were subsequently harvested at the indicated time points, and protein was extracted using NP-40 lysis buffer. Cell lysate was resolved on SDS-PAGE using Novex BOLT 4 to 12% (Thermo Scientific) gradient gel using MES [2-(*N*-morpholino)ethanesulfonic acid] buffer (Thermo Scientific). Proteins were subsequently transferred to a polyvinylidene difluoride (PVDF) membrane using Genescript’s eBLOT L1 with default settings, and the membranes were blotted using the previously described antibodies. Detection was performed using secondary HRP-conjugated antibodies and exposure to radiographic film.

### Real-time gene expression analysis.

Real-time gene expression was performed as previously described (20, 21). Briefly, 293 cells were infected in triplicate with each indicated viral construct at an MOI of 10 vp/cell for 24 h. Total RNA was extracted using TRI reagent (MilliporeSigma) according to the manufacturer’s instructions. Any remaining genomic DNA was removed using an Invitrogen TURBO DNA-free kit. RNA yields were quantified via spectrophotometry (Molecular Devices SpectraMax iD3) and 1 μg from each sample was used to generate cDNA with SuperScript IV VILO master mix (Invitrogen) according to the manufacturer’s guidelines. This cDNA was subsequently used for real-time expression analysis with the Bio-Rad CFX96 real-time thermal cycler and Applied Biosystems SYBR select master mix for CFX, with an annealing step of 60°C for 1 min. Expression data were normalized to glyceraldehyde-3-phosphate dehydrogenase (GAPDH) mRNA levels.

### Immunoprecipitation, SDS-PAGE, and silver staining analysis.

The 293 cells were plated onto 15-cm plates and allowed to grow until confluent and then infected with each indicated viral construct at an MOI of 100 vp/cell. Then, 24 h after infection, cells were harvested and lysed in 1.5 mL of NP-40 lysis buffer. Cell nuclei were pelleted via centrifugation, and 950 μL of supernatant was transferred to a new 1.5-mL tube. Cell lysates were then incubated with 30 μL of anti-HA affinity matrix (anti-rat IgG, clone 3F10). Samples were then washed and run on a 10% SDS-PAGE hand cast gel at 30 V for 16 h and then finished at 200 V for 2 h in a Bio-Rad Protean II cell. The protein was visualized via silver staining as per the manufacturer’s protocol (Invitrogen SilverQuest silver-staining kit) according to the manufacturer’s protocol.

### Immunofluorescence.

Cell staining was performed as previously described (22). Briefly, HT-1080 cells were plated at low density (20,000 cells per chamber) on chamber slides (Nalgene Nunc) and subsequently infected with each rHAdV at an MOI of 1,000 vp/cell. Then, 24 h after infection, cells were fixed in 4% formaldehyde, blocked in blocking buffer (5% bovine serum albumin [BSA], 0.2% Tween 20 in phosphate-buffered saline [PBS]), and stained with either primary antibody 3F10 or 2B3E5, followed by the corresponding Alexa Fluor 594-tagged secondary antibodies. After staining and extensive washing with PBS with Tween 20 (PBST), slides were mounted using Prolong Diamond antifade mountant with DAPI (4′,6-diamidino-2-phenylindole; Invitrogen) and imaged using a Zeiss LSM700 confocal laser scanning microscope. Images were analyzed using the Zeiss ZEN software package.
